# Overexpression of small-conductance Ca^2+^-activated K^+^ channel 2 attenuates pain-like behavior in female mice with cystitis

**DOI:** 10.1172/jci.insight.199567

**Published:** 2026-05-22

**Authors:** Guadalupe Manrique-Maldonado, Xuejiao Sun, Allison L. Marciszyn, Nicolas Montalbetti, Marcelo D. Carattino

**Affiliations:** 1 Renal-Electrolyte Division, Department of Medicine, and; 2 Department of Cell Biology, University of Pittsburgh, Pittsburgh, Pennsylvania, USA.

**Keywords:** Nephrology, Neuroscience, Pain, Urology

## Abstract

Small-conductance Ca^2+^-activated K^+^ (SK) channels regulate neuronal excitability and act as a feedback mechanism to limit firing during sustained stimulation. In the present study, we demonstrated that SK2 plays an important role in the control of bladder function and visceral pain processing. SK2 channels are expressed in bladder-innervating afferent neurons, and ablation of this subunit results in elevated afferent firing rates in response to physiological levels of bladder distension, supporting a role for SK2 in modulating mechanosensory excitability. Mice overexpressing SK2 exhibit increased bladder capacity and reduced voiding frequency. Furthermore, overexpression of SK2 prevents the onset of pelvic mechanical allodynia and attenuates the exaggerated visceromotor response to bladder distension seen in wild-type mice with chemical cystitis. Thus, SK2 may be a promising target for treating overactive bladder and pain originating from the urinary bladder and other pelvic organs.

## Abstract 2

Small-conductance Ca^2+^-activated K^+^ channel 2 may be a promising target for treating overactive bladder and pain originating from the urinary bladder and other pelvic organs.

## Graphical abstract

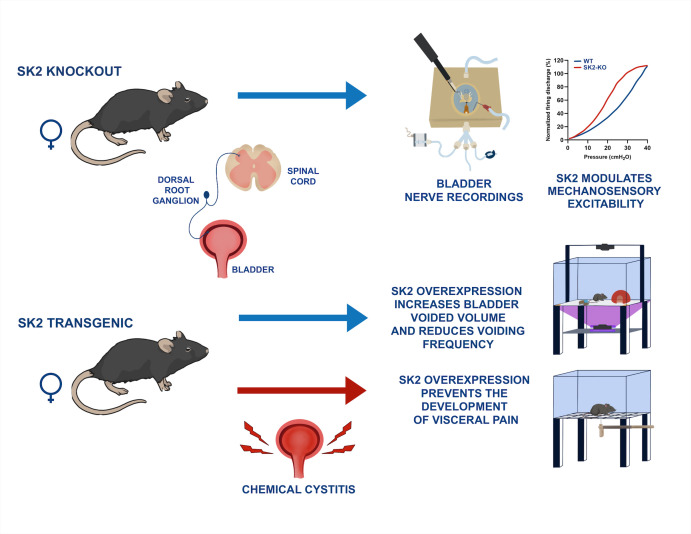

## Introduction

Visceral pain is a common and debilitating condition that occurs in certain disease processes that affect internal organs, and it imposes a social burden on patients and enormous economic cost in health systems and society (1). Unlike somatic pain, which affects muscles, bones, and soft tissues, visceral pain is described as vague, diffuse, and poorly localized, and it is referred to other locations via motor or autonomic reflexes (2–4). Pain is a complex entity that evolves as it transitions from the acute to the chronic phase, each controlled by different mechanisms. Although the clinical management of visceral and somatic pain is in general similar, the neurological mechanisms, the activation of peripheral and central pathways, and the perception and psychological processing as well as the progression of visceral pain differ from those of somatic pain (5, 6). Medications used to treat visceral pain are, for the most part, inadequate at relieving symptoms, and their use is frequently constrained by side effects (5–7). Therefore, finding new targets is crucial for developing effective treatments for visceral pain conditions.

Visceral pain is a direct consequence of the sensitization of neuronal pathways (i.e., primary sensory neurons and spinal ascending neurons) involved in the transmission of information from internal organs to the supraspinal level. Alternatively (or in addition), it can arise from the dysregulation of descending pathways that modulate spinal nociceptive transmission (4, 8–10). Small-conductance calcium-activated K^+^ (SK) channels are an attractive target for treating pain, because they are activated exclusively by rises in intracellular Ca^2+^ levels, such as those occurring in neurons during periods of high activity (e.g., sensitization) (11).

In a wide variety of central neurons, SK channels mediate the afterhyperpolarization phase of the action potential and control the frequency of firing and adaptation (11). Although SK channels have been described in sensory neurons and central neurons (12–14), their role in nociception remains largely unexplored. Three homologous pore-forming SK subunits have been cloned, SK1, SK2, and SK3, that associate to form homo- or heterotetrameric channels that exist in a stable complex with calmodulin (15–17). The activation of SK channels is initiated by the binding of Ca^2+^ to calmodulin, which triggers a conformational change in the pore-forming subunits that opens the conductive pathway (18). Here we use genetic tools to examine the role of SK2 channels in normal bladder function and nociception during chemical cystitis.

## Results

### SK2 is expressed in bladder sensory neurons.

**Figure 1:**
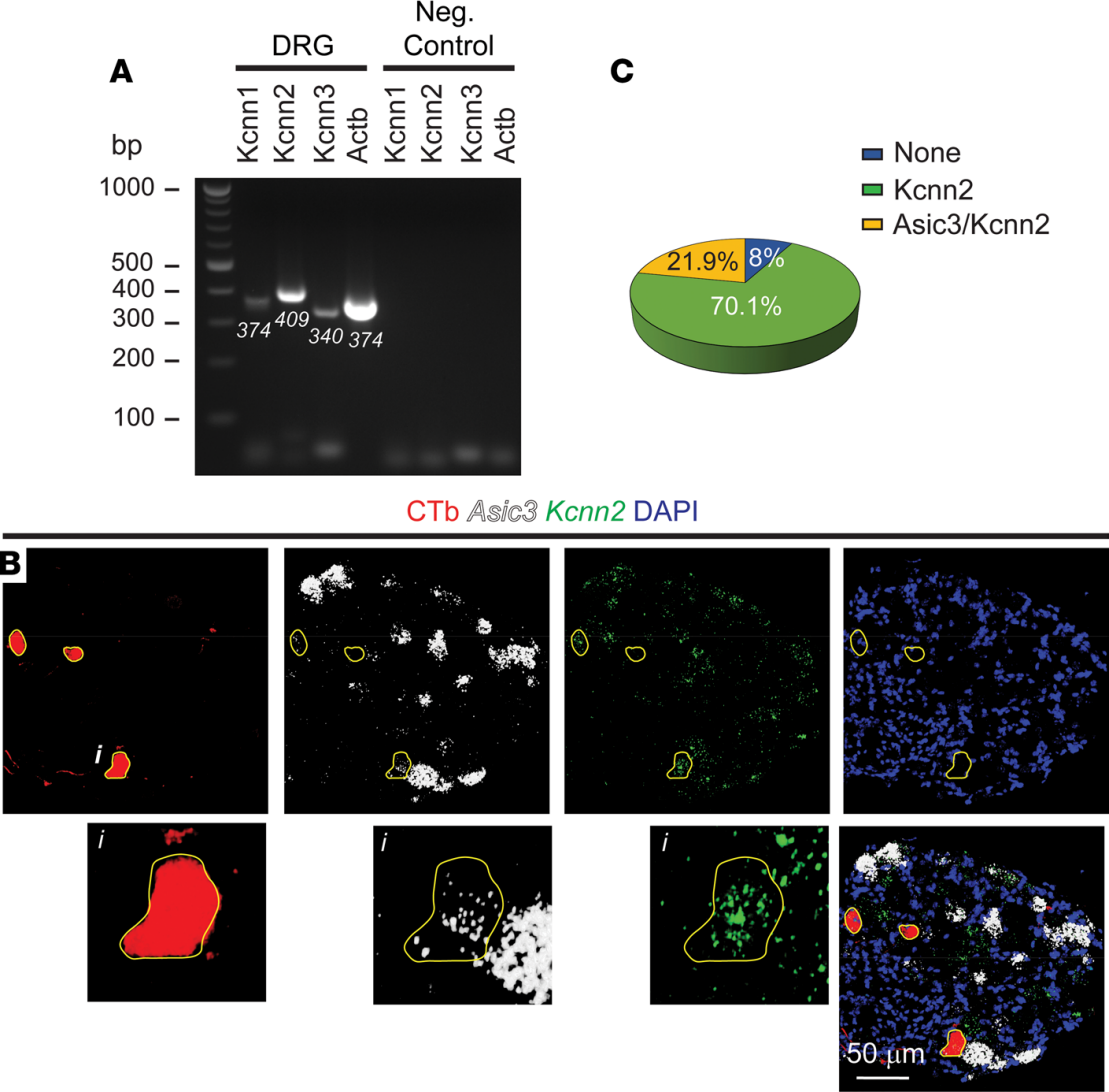
SK2 channels are expressed in bladder sensory neurons. (**A**) RT-PCR confirms the expression of transcripts for SK1 (*Kcnn1*), SK2 (*Kcnn2*), and SK3 (*Kcnn3*) in dorsal root ganglia (DRG) (lines 2–5). Actin (*Actb*) served as a positive control for reverse transcription and amplification. Expected PCR product sizes are shown. Negative control reactions included GoTaq, primers, and DMSO, but not sample (lines 6–9). Representative of 3 experiments. (**B**) *Kcnn2* is expressed in bladder-innervating DRG neurons. Immuno-FISH was performed in fresh-frozen sections of DRG (L6–S1) harvested from mice injected into the bladder wall with cholera toxin β subunit (CTb) conjugated to Alexa Fluor 555. A rabbit anti-CTb antibody and a secondary goat anti-rabbit antibody conjugated with Cy3 were used to identify bladder sensory neurons (red). Bottom: 4-fold magnification of a CTb-labeled neuron in box (*i*). No signal was visible with the negative control probe (not shown). Scale bar: 50 μm. (**C**) Quantitative analysis of expression for *Kcnn2* and *Asic3* in bladder sensory neurons. Percentage of neurons expressing *Kcnn2*, or *Kcnn2* and *Asic3*, is shown (*n* = 187 bladder-innervating neurons from 8 L6–S1 DRG from 4 mice).

Sensory information from the urinary bladder related to fullness as well as pain is carried out by afferent fibers in the pelvic and hypogastric nerves (19). The cell bodies of these sensory neurons reside in dorsal root ganglia (DRG) at the thoracolumbar (i.e., hypogastric nerve afferents) and lumbosacral (i.e., pelvic nerve afferents) levels, and their peripheral terminals are distributed throughout the bladder mucosa and muscularis externa (19). To determine whether SK channel subunits are expressed in sensory neurons, we isolated mRNA from lumbosacral (L6–S2) DRG of naive mice and performed reverse transcription followed by PCR amplification with primers targeting SK1 (*Kcnn1*), SK2 (*Kcnn2*), and SK3 (*Kcnn3*). Actin (*Actb*) served as a control for the reverse transcription and amplification steps. A prominent band of the expected molecular size for *Kcnn2* was amplified from RNA purified from lumbosacral DRG (Figure 1A). Although PCR products of expected size were amplified with primers for *Kcnn1* and *Kcnn3*, the bands were barely visible (Figure 1A). Thus, we centered subsequent studies on *Kcnn2*. To confirm that *Kcnn2* is expressed in the DRG neurons that innervate the urinary bladder, we combined retrograde tracing with the cholera toxin β subunit (CTb) and immunofluorescence in situ hybridization (immuno-FISH). CTb conjugated to Alexa Fluor 555 was injected into the bladder wall, and after 7–10 days, during which time the tracer was transported retrogradely along the axons, L6–S2 DRG were harvested. As the fluorescent signal of the CTb-labeled neurons was weak, a primary antibody raised against CTb was used to detect labeled neuron in immuno-FISH studies. *Asic3*, which encodes a subunit of a proton-gated channel expressed in bladder sensory neurons, was used as control in these studies (20). We found that the majority of DRG sensory neurons labeled with CTb expressed *Kcnn2* (92%) (Figure 1, B and C).

**Figure 2:**
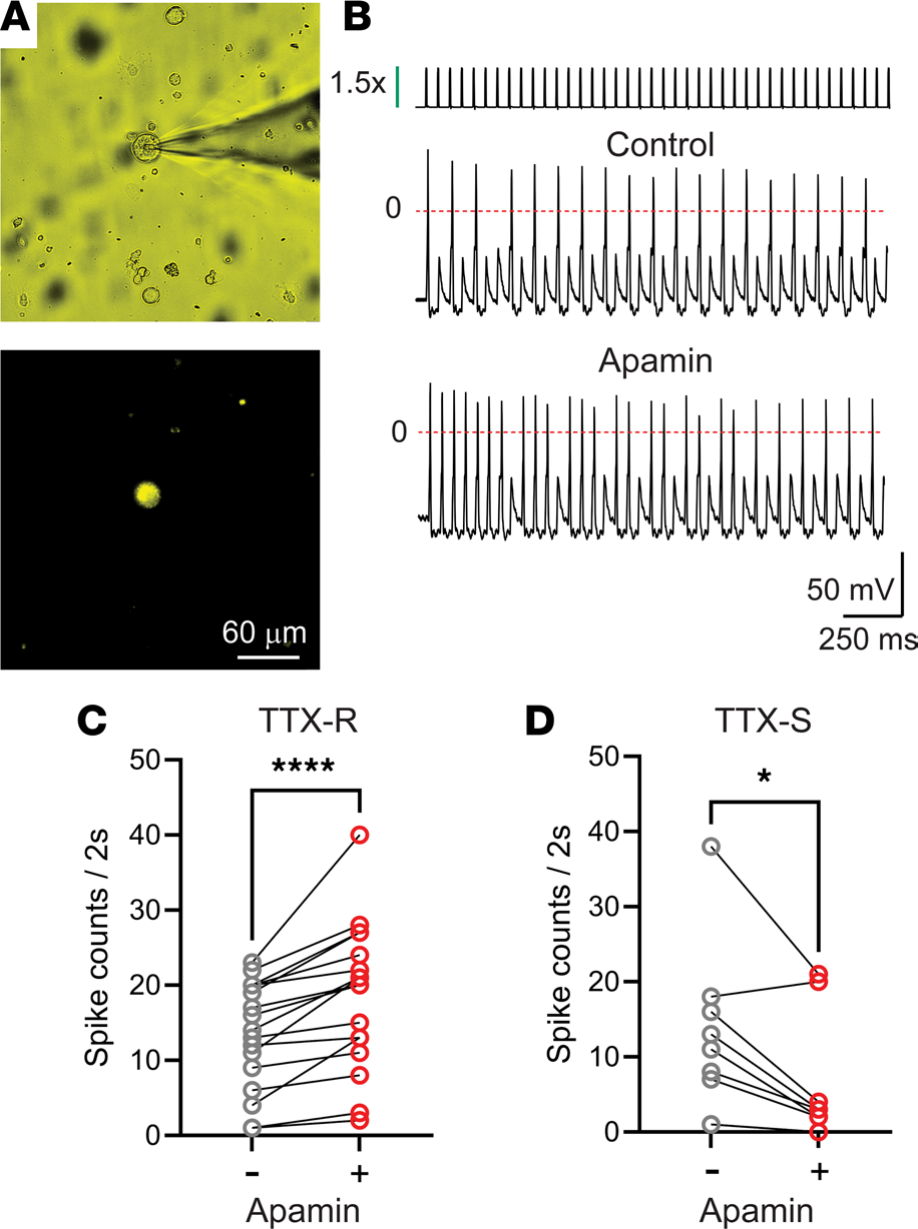
SK channels regulate DRG neuron firing. (**A**) Bright-field (top) and epifluorescence (bottom) images of isolated DRG neurons (L6–S2) captured during a patch-clamp experiment. Scale bar: 60 μm. (**B**) Apamin increases the firing frequency of bladder sensory neurons with tetrodotoxin-resistant (TTX-R) action potential. Neuronal firing was evoked by suprathreshold current pulses with a width of 4 ms and an amplitude equal to 1.5 times the rheobase at a rate of 20 Hz. The stimulation protocol is shown on the top. (**C** and **D**) Number of spikes discharged in response to electrical stimulation for bladder sensory neurons with TTX-R (**C**) and TTX-sensitive (TTX-S) (**D**) action potentials. The number of spikes discharged before and in the presence of apamin (100 nM) is shown (TTX-R, *n* = 17; TTX-S, *n* = 9; *P < 0.05, ****P < 0.0001, Wilcoxon’s matched-pairs non-parametric signed-rank test).

To assess whether SK channels regulate bladder sensory neuron excitability, we examined their firing under control conditions and in the presence of apamin (100 nM), a specific inhibitor of SK1, SK2, and SK3 channels. To label bladder sensory neurons, the lipophilic membrane stain DiI (1,1′-dioctadecyl-3,3,3′,3′-tetramethylindocarbocyanine perchlorate) was injected into the bladder wall. Seven to ten days later, L6–S2 DRG were isolated and enzymatically dissociated. Retrogradely labeled bladder sensory neurons were studied with the patch-clamp technique (Figure 2A). Neuronal firing was evoked by suprathreshold current pulses with a width of 4 ms and an amplitude equal to 1.5 times the rheobase (i.e., minimal current necessary to trigger an action potential) at a rate of 20 Hz. The number of action potentials evoked in response to electrical stimulation in the absence and presence of the SK channel inhibitor apamin (100 nM) was computed (Figure 2, B–D). Bladder sensory neurons were classified according to the sensitivity of the action potential to 1 μM tetrodotoxin (TTX), as TTX-sensitive (TTX-S) or TTX-resistant (TTX-R) (20–25). The mean resting potential for neurons with TTX-R and TTX-S action potentials was –62.49 ± 2.2 (*n* = 17) and –57.3 ± 2.3 (*n* = 9), respectively. Apamin increased the firing frequency of neurons exhibiting TTX-R action potentials (Figure 2C), but unexpectedly, it caused a reduction in the number of spikes evoked by train stimulation in bladder neurons with action potentials inhibitable by TTX (Figure 2D). In summary, these results indicate that SK channels are expressed in the sensory neurons that innervate the urinary bladder, where they control action potential discharge during high-frequency stimulation.

### SK2 controls the firing of bladder afferents.

**Figure 3:**
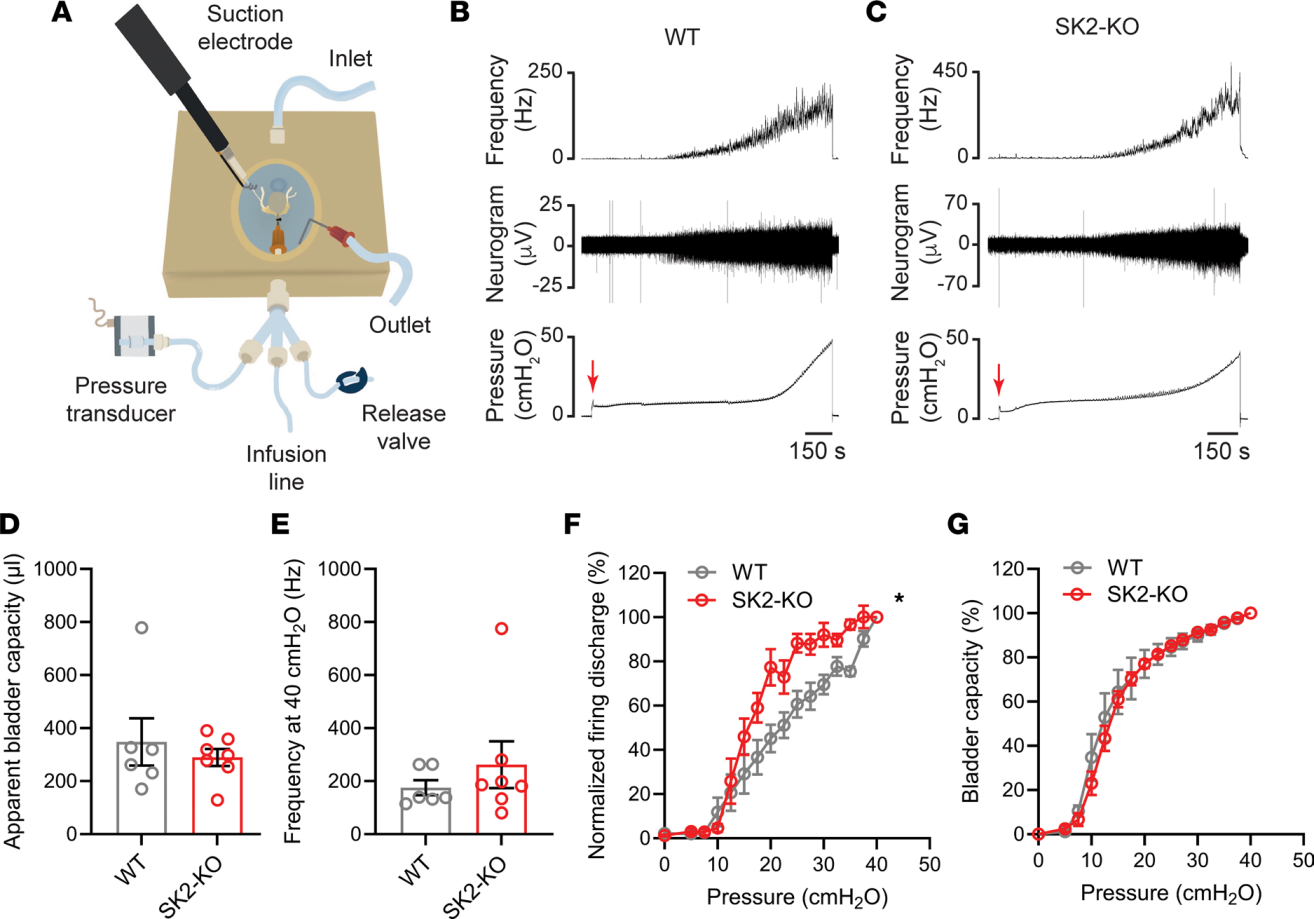
SK2 regulates bladder afferent firing. Intravesical pressure and afferent discharge were recorded simultaneously during continuous filling from an ex vivo preparation. (**A**) Diagram of the nerve recording chamber and recording setup. (**B** and **C**) Representative recordings of intravesical pressure, raw nerve activity, and afferent discharge from a bladder of a naive control (**B**) and an SK2-KO mouse (**C**). Bladder infusion initiation is denoted by a red arrow. (**D**) Apparent bladder capacity in SK2-KO and WT littermate mice. Urinary bladders were infused at a rate of 15 μL/min until the intravesical pressure reached 40 cmH_2_O. Apparent bladder capacity represents the volume required to increase the intravesical pressure to 40 cmH_2_O. (**E**) Mean afferent nerve discharge frequency at 40 cmH_2_O for SK2-KO and WT littermate mice. (**F**) Normalized afferent discharge as a function of intravesical pressure for bladders of WT and SK2-KO mice. Data are shown as the mean ± SEM (WT, *n* = 6; SK2-KO, *n* = 7; 2-way ANOVA, *P < 0.05). (**G**) Bladder capacity as a function of intravesical pressure for bladders of WT and SK2-KO mice. No difference in bladder capacity versus pressure was observed between WT and SK2-KO.

To investigate the function of SK2 in afferent signaling, we performed nerve recordings from bladders of SK2-knockout (SK2-KO) and wild-type (WT) littermates. The bladder, urethra, and associated pelvic nerve roots were harvested, and a catheter was implanted in the urethra for infusion. The preparation was transferred to a temperature-regulated chamber, and afferent activity was registered with a suction electrode during continuous bladder filling as described previously (20, 26–28) (Figure 3A). Urinary bladders were continuously infused at a rate of 15 μL/min until the intravesical pressure reached approximately 40 cmH_2_O (Figure 3, B and C). Apparent bladder capacity and mean firing frequency rate, calculated at an intravesical pressure of 40 cmH_2_O, were similar in control and SK2-KO mice (Figure 3, D and E). In both SK2-KO and WT littermates, bladder afferents began to discharge above basal levels when the intravesical pressure reached approximately 10 cmH_2_O (Figure 3F). However, normalized firing discharge increased faster as the intravesical pressure rose in SK2-KO than in WT controls (Figure 3F). To assess whether the loss of SK2 affects the ability of the urinary bladder to accommodate fluid during the filling, we plotted bladder capacity as a function of the intravesical pressure (Figure 3G). No difference was observed in the bladder capacity/pressure relationship between the 2 groups, indicating that bladder wall mechanics and elasticity were similar in controls and SK2-KO mice. These results indicate that SK2 channels regulate the excitability of bladder afferents. SK2-null mice are smaller than WT mice and develop whole-body tremor with ataxia and impaired righting reflex within 2 weeks of age (29, 30). This limited our ability to assess voiding behavior in SK2-KO mice and to study the role of SK2 in visceral sensation.

### Kcnn2 expression in the bladder and DRG of SK2^+/T^ mice.

**Figure 4:**
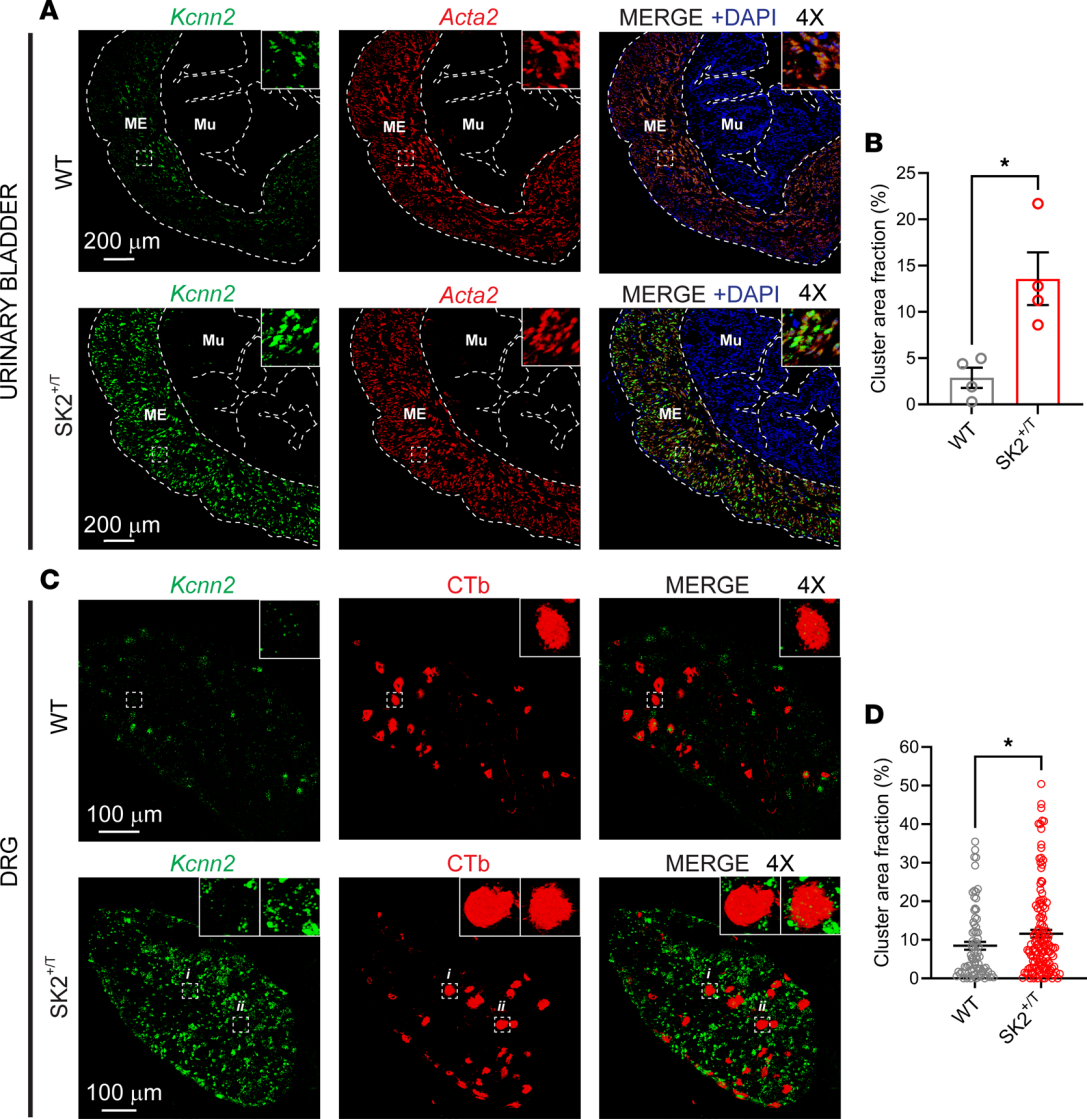
Overexpression of *Kcnn2* in transgenic SK2 mice. FISH and immuno-FISH confirm higher *Kccn2* expression in tissues of SK2^+/T^ mice than control littermates (WT). No signal was visible with the negative control probe (not shown). (**A**) Confocal images of the urinary bladder. FISH was performed with probes for *Kcnn2* and *Acta2*. *Acta2* is highly expressed in smooth muscle cells. Insets: 4-fold magnification of the muscularis externa. Note the overlap between *Kcnn2* and *Acta2*. Mu, mucosa; ME, muscularis externa. Scale bars: 200 μm. (**B**) Quantification of *Kcnn2* expression in the bladder muscularis externa. The fraction area of the muscularis externa occupied by *Kcnn2* clusters is shown. Data are shown as the mean ± SEM (WT, *n* = 4; SK2^+/T^, *n* = 4; Mann-Whitney test, *P < 0.05). (**C**) Confocal images of DRG. Immuno-FISH was performed in fresh-frozen sections of DRG (L6–S2) harvested from mice injected into the bladder wall with cholera toxin β subunit (CTb). A goat anti-CTb antibody and a secondary donkey anti-goat antibody conjugated with Alexa Fluor 594 were used to identify bladder sensory neurons (red). Insets: 4-fold magnification of CTb-labeled neurons. Scale bars: 100 μm. (**D**) Quantification of *Kcnn2* expression in CTb-labeled neurons. The fraction area of each CTb-labeled neuron occupied by *Kcnn2* clusters is shown. Data are shown as the mean ± SEM (WT, 78 cells from 2 mice; SK2^+/T^, 133 cells from 2 mice; Mann-Whitney test, *P < 0.05).

Our studies indicate that the loss of SK2 results in increased afferent discharge at physiological bladder distension pressures. Thus, to assess whether SK2 gain of function could be used to treat visceral hypersensitivity, we obtained heterozygous transgenic mice that overexpress *Kcnn2* as a result of the insertion of a tetracycline-based genetic switch before the translation initiation site of this gene (31). To confirm that SK2^+/T^ mice overexpress *Kcnn2* in tissues that inherently express the gene, we performed FISH in DRG and bladders obtained from transgenic mice and WT littermates (Figure 4). *Kcnn2* RNA transcripts, which appear as puncta and clusters in these images, were detected in bladder smooth muscle cells of WT mice that also expressed smooth muscle actin (*Acta2*) (Figure 4A). No other cell type in the bladder wall appeared to express *Kcnn2*. *Kcnn2* expression was increased 4.72-fold in the bladder muscularis externa of SK2^+/T^ mice (Figure 4B). *Kcnn2* expression was greater in whole DRG of SK2^+/T^ mice than controls (Figure 4C). However, quantitative analysis revealed only a small increase in *Kcnn2* expression in L6–S1 *Calca^+^* DRG neurons (0.78-fold) (Supplemental Figure 1; supplemental material available online with this article; https://doi.org/10.1172/jci.insight.199567DS1) and in bladder-innervating DRG neurons (0.37-fold) of SK2^+/T^ mice when compared with those of control mice (Figure 3, C and D). Thus, our data indicate that *Kcnn2* expression is increased in cell types other than neurons in the DRG of SK2^+/T^ mice. In summary, these findings indicate that *Kcnn2* is not evenly overexpressed across tissues in SK2^+/T^ mice.

### Kcnn2 overexpression limits visceral nociceptive responses in mice with chemical cystitis.

In the lower urinary tract, pain arises with inflammatory processes that affect the bladder wall, including urinary tract infections, interstitial cystitis/bladder pain syndrome, cancer, pelvic radiation therapy, and exposure to chemicals (e.g., cyclophosphamide) (24, 32–35). To investigate the role of SK2 in bladder nociception, we used a chronic model of chemical cystitis induced by cyclophosphamide (CYP) (36, 37). This alkylating agent is clinically used to treat an array of cancers and as an immune suppressor in nephrotic syndrome, in granulomatosis with polyangiitis, and following organ transplantation (34). CYP is metabolized in the liver to acrolein, a highly reactive aldehyde, which induces cystitis as it accumulates in the urinary bladder (32, 38). Although MESNA, a thiol compound, is usually administered with CYP as a uroprotective agent, a significant number of patients treated with these agents still develop cystitis and cancer (39). We and others have shown that mice treated with 4 doses of CYP (80 mg/kg) on alternate days for a week exhibit increased voiding frequency with voids of small volume and pelvic allodynia (36, 37).

**Figure 5:**
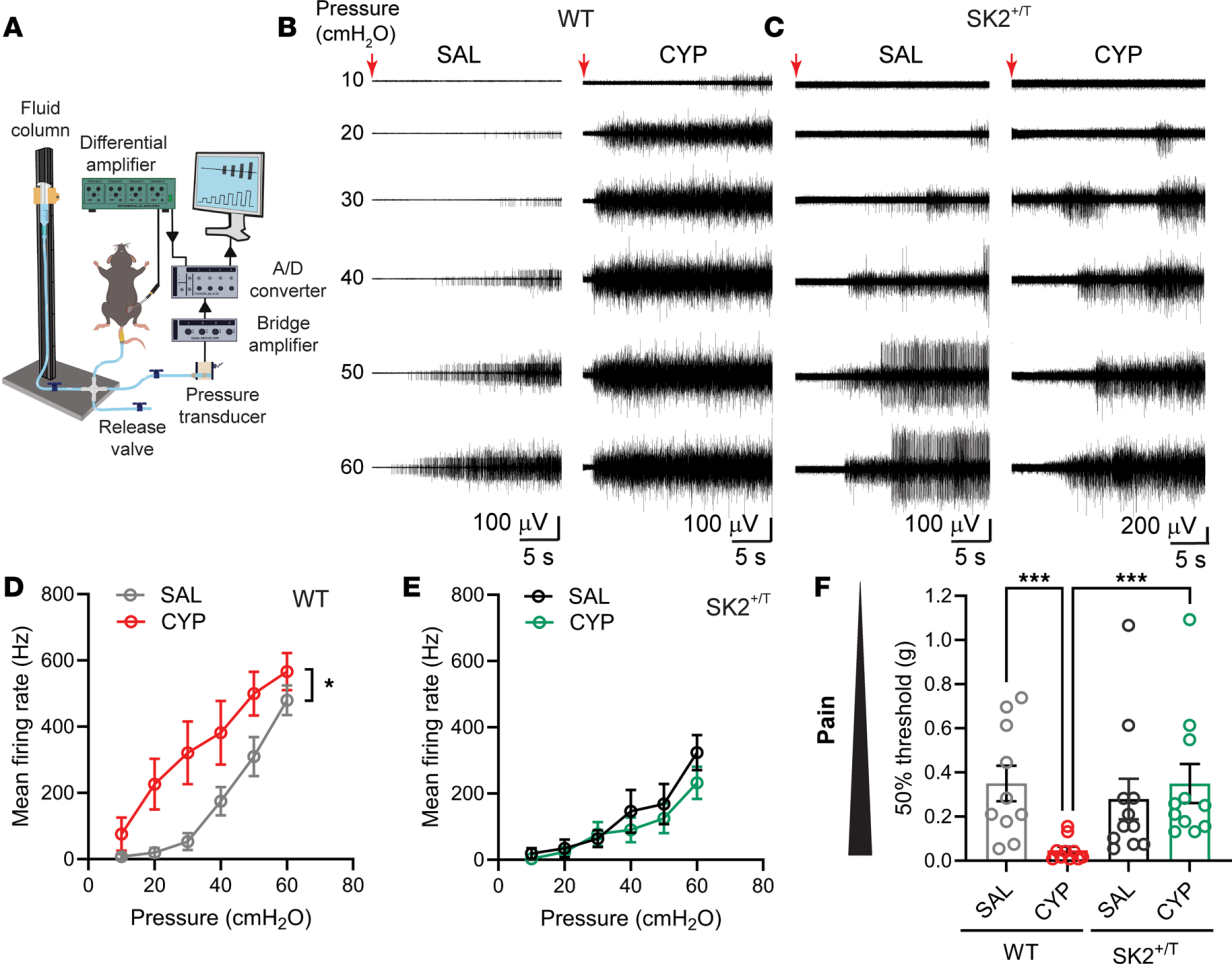
SK2 overexpression attenuates nociceptive responses in mice with chemical cystitis. (**A**) Experimental setup to assess the visceromotor response (VMR) to bladder distension. Saline is infused into the urinary bladder using a urethral catheter. Electromyography (EMG) activity is recorded with electrodes implanted in the oblique muscle and intravesical pressure with a transducer connected to the urethral catheter. (**B** and **C**) Representative EMG tracings during bladder distension for WT (**B**) and SK2^+/T^ (**C**) mice treated with saline (SAL) and cyclophosphamide (CYP). Intravesical pressure is indicated on the left. SAL or CYP was administered every other day for a week. Bladder infusion initiation is denoted by a red arrow. (**D**) CYP administration potentiates the VMR response to bladder distension in WT mice (WT-SAL, *n* = 10; WT-CYP, *n* = 9; 2-way ANOVA, *P < 0.05). (**E**) SK2^+/T^ mice are unaffected by CYP treatment (SK2^+/T^-SAL, *n* = 7; SK2^+/T^-CYP, *n* = 6). (**F**) Fifty percent withdrawal threshold grams to von Frey filaments applied to the pelvic area for WT and SK2^+/T^ mice treated with SAL or CYP (WT-SAL, *n* = 10; WT-CYP, *n* = 10; SK2^+/T^-SAL, *n* = 11; SK2^+/T^-CYP, *n* = 11; ***P < 0.001, Kruskal-Wallis test followed by Dunn’s multiple-comparison test). Data are shown as the mean ± SEM.

To determine whether CYP alters *Kcnn2* expression in the urinary bladder and/or DRG, we performed FISH (i.e., bladder) and immuno-FISH (i.e., DRG) in tissues harvested from WT mice injected with saline or CYP. No difference in *Kcnn2* expression was observed between the 2 groups (Supplemental Figure 2). To assess visceral sensation, we measured the visceromotor response (VMR), a contraction of the abdominal musculature evoked by bladder distension, in SK2^+/T^ mice and WT littermates treated with saline or CYP. The VMR is a visceral reflex arc that involves visceral afferents, a spinal integrating center, efferent neurons, and abdominal muscles and is triggered in response to noxious stimuli applied to internal organs. To record electromyography activity, electrodes were implanted in the oblique muscles, and the urethra was catheterized to facilitate bladder filling (Figure 5A). After a period of equilibration, the urinary bladder was infused with saline solution to defined pressures (10, 20, 30, 40, 50, and 60 cmH_2_O) for 30 seconds with 3-minute intervals between distensions. In WT mice that received saline, motor units in oblique muscles began to discharge when the intravesical pressure reached 20–30 cmH_2_O (Figure 5B). CYP treatment reduced the pressure threshold for the VMR in WT mice and caused robust firing at physiological pressures (i.e., <30 cmH_2_O) (Figure 5, B and D). Note that the mean firing rate (Hz) at 60 cmH_2_O was not different between WT mice injected with saline (479.7 ± 45.01, *n* = 10) versus CYP (566.3 ± 56.34, *n* = 9) (Figure 5D). In contrast, the VMR to bladder distension was not different between SK2^+/T^ mice that received saline versus CYP (Figure 5, C and E). These findings suggest that SK2 channels modulate the transmission of nociceptive information originating from the bladder in the context of chemically induced cystitis.

To further assess the effect of SK2 overexpression on visceral nociception, we evaluated somatic pelvic sensitivity in awake mice with von Frey filaments using the up-down method described by Chaplan et al. (40) (Figure 5F). Note that the lower the 50% threshold to evoke a response by von Frey filaments, the greater the pelvic sensitivity to mechanical stimuli. In good agreement with our previous studies (40), WT mice treated with CYP exhibited increased pelvic sensitivity (i.e., allodynia) to von Frey filaments when compared with controls that received saline (Figure 5F). SK2^+/T^ mice did not develop pelvic allodynia when treated with CYP. Lastly, to assess whether the beneficial effects of SK2 overexpression on visceral sensitivity are due to the overexpression of the channel in the abdominal skeletal muscle and a change in muscle tone, we performed FISH with samples of abdominal skeletal muscle from control and SK2^+/T^ mice. *Kccn1a*, which encodes the pore-forming subunit of the large-conductance calcium-activated K^+^ channel and is expressed in skeletal muscle, served as a positive control for these experiments. Bladder tissues were processed in parallel and served as positive control. We found that the expression of *Kcnn2* in skeletal muscle was insignificant (Supplemental Figure 3A). Images of bladder samples from control and SK2^+/T^ mice run in parallel are shown (Supplemental Figure 3B). Thus, the beneficial effects of *Kcnn2* overexpression on visceral sensitivity and pelvic allodynia in mice treated with CYP cannot be attributed to changes in skeletal muscle tone. Taken together, our data indicate that the overexpression of SK2 suppresses visceral nociceptive responses in mice with chemical cystitis.

### SK2 overexpression alters voiding behavior.

**Figure 6:**
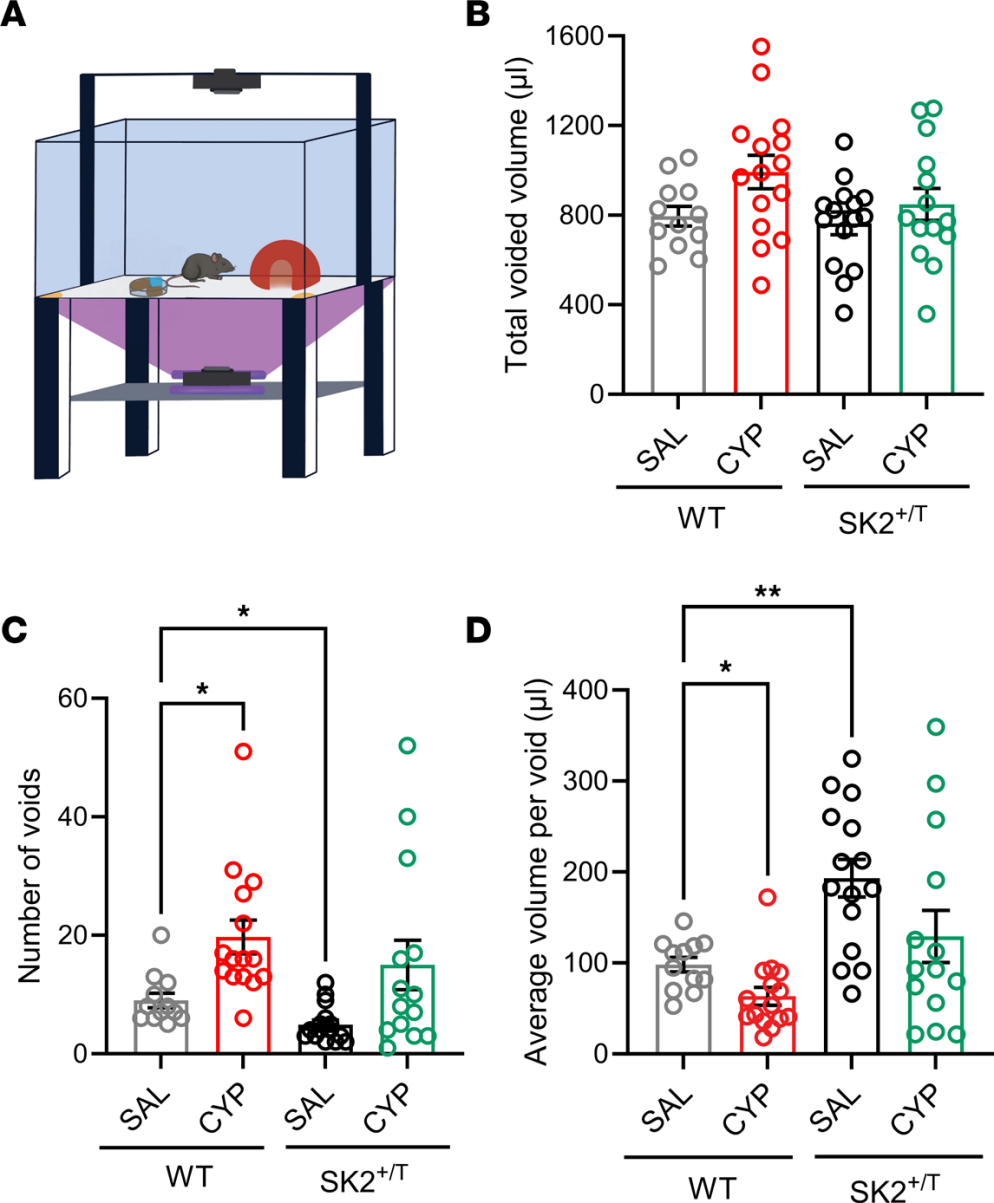
Voiding behavior of transgenic SK2 mice. Voiding activity was evaluated over a 12-hour time window during the dark phase in freely mobile SK2^+/T^ and WT littermate mice. Saline (SAL) or CYP was administered every other day for a week. (**A**) Diagram of the system used to assess voiding behavior in awake mice. A filter paper is placed on the bottom of the chamber housing the mouse. The chamber is illuminated from below with UV light, allowing the visualization of the urine spots. Activity is recorded using the top and bottom cameras. (**B**) Total voided volume during a 12-hour period. (**C**) Number of voids during a 12-hour period. (**D**) Average volume per void. Data are shown as the mean ± SEM (WT-SAL, *n* = 12; WT-CYP, *n* = 15; SK2^+/T^-SAL, *n* = 15; SK2^+/T^-CYP, *n* = 14; *P < 0.05, **P < 0.01, Brown-Forsythe and Welch’s ANOVA test followed by Dunnett’s T3 multiple-comparison test).

To evaluate conscious voiding behavior in freely moving female mice, we used a video-monitored void spot assay with fluorescence-based detection of urine spots (41) (Figure 6A). This system allows us to measure urinary frequency and void volumes with accuracy during long periods of time (>12 hours). SK2^+/T^ and WT littermate mice were treated with saline or CYP for a week as indicated above. Voiding behavior was evaluated during the dark cycle (active phase) a day after mice received the last dose of saline or CYP. The total voided volume over the 12 hours of the dark cycle was similar for all the groups (Figure 6B). However, SK2^+/T^ mice treated with saline tended to void larger volumes and less often than WT littermates, consistent with SK2 overexpression increasing bladder capacity (Figure 6, C and D). As expected, WT mice treated with CYP exhibited increased urinary frequency and voided smaller volumes compared with controls that received saline. Even though SK2^+/T^ mice treated with CYP urinated overall more times than those that received saline, the difference was not statistically significant (Figure 6C). The average volume per void was similar between SK2^+/T^ that received saline versus CYP (Figure 6D). In summary, SK2^+/T^ mice void larger volume and less frequently than controls. SK2 overexpression partially reverses the effects of CYP on voiding seen in WT littermate mice.

### Chronic CYP treatment and afferent function.

**Figure 7:**
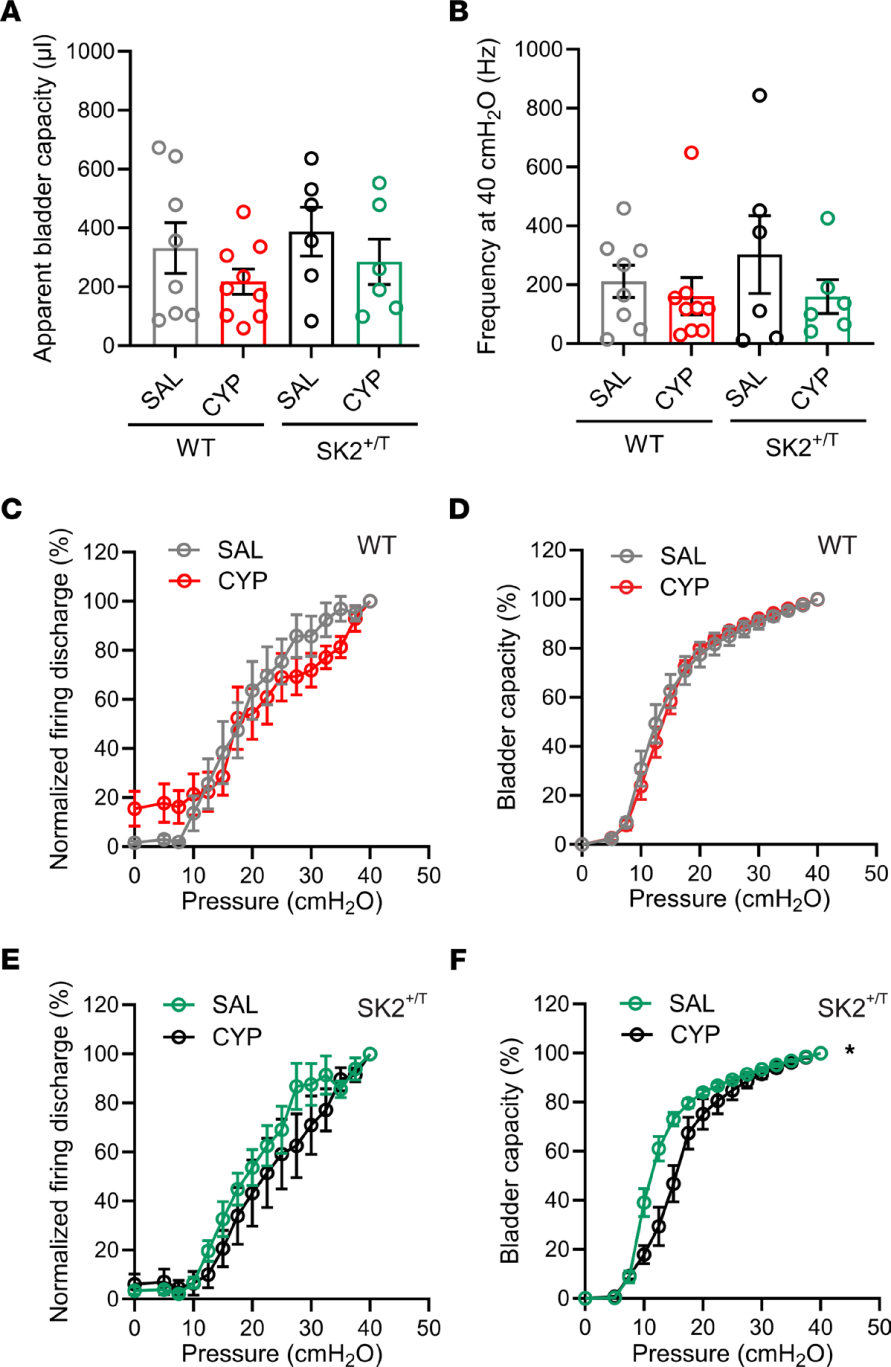
Chronic cystitis and afferent function. (**A**) Apparent bladder capacity for WT and SK2^+/T^ mice treated with saline (SAL) or cyclophosphamide (CYP). Urinary bladders were infused at a rate of 15 μL/min until the intravesical pressure reached 40 cmH_2_O. Apparent bladder capacity represents the volume required to increase the intravesical pressure to 40 cmH_2_O. (**B**) Mean afferent nerve discharge frequency at 40 cmH_2_O for WT and SK2^+/T^ mice treated with SAL or CYP. (**C** and **E**) Normalized afferent discharge as a function of intravesical pressure for bladders of WT (**C**) and SK2^+/T^ mice (**E**) treated with SAL or CYP. No difference in bladder afferent discharge was observed between mice treated with SAL versus CYP. Data are expressed as the mean ± SEM (WT-SAL, *n* = 8; WT-CYP, *n* = 9; SK2^+/T^-SAL, *n* = 6; SK2^+/T^-CYP, *n* = 6). (**D** and **F**) Bladder capacity as a function of intravesical pressure for bladders of WT (**D**) and SK2^+/T^ mice (**F**) treated with SAL or CYP. Data are shown as the mean ± SEM (WT-SAL, *n* = 8; WT-CYP, *n* = 9; SK2^+/T^-SAL, *n* = 6; SK2^+/T^-CYP, *n* = 6; 2-way ANOVA, *P < 0.05).

Bladder afferents are prone to sensitization by chemicals, proinflammatory molecules, and bacterial products (3, 4, 24, 42). To assess whether the visceral hypersensitivity seen in WT mice treated with CYP was driven by sensitized afferents, we performed multi-unit afferent nerve recordings from urinary bladders of mice injected with saline or CYP (Figure 7 and Supplemental Figure 4). Apparent bladder capacity and mean firing frequency rate, calculated at an intravesical pressure of 40 cmH_2_O, were similar in control and SK2^+/T^ mice treated with saline or CYP (Figure 7, A and B). As expected, in both WT and SK2^+/T^ mice treated with saline, bladder afferents began to discharge above basal levels when the intravesical pressure reached approximately 10 cmH_2_O (Figure 7, C and E). Although basal afferent activity was slightly higher in WT mice treated with CYP than in controls treated with saline, the difference was not statistically significant. Unexpectedly, CYP treatment did not alter afferent firing in either group (Figure 7, C and E). To determine whether CYP affects the ability of the urinary bladder to accommodate fluid during the filling phase, we plotted normalized bladder capacity as a function of the intravesical pressure (Figure 7, D and F). For WT mice, the relationship between bladder capacity and pressure during the filling phase was similar independently of the treatment that they received. In contrast, bladders from SK2^+/T^ mice treated with CYP required a larger fractional volume to reach 50% bladder capacity than those of transgenic mice that received saline. This finding suggests that CYP treatment alters bladder wall mechanics or elasticity in this group.

## Discussion

Ca^2+^-activated K^+^ channels are engaged during action potential repolarization (i.e., large-conductance Ca^2+^-activated K^+^ [BK] channels) and afterhyperpolarization (SK channels) to provide feedback inhibition and regulate action potential firing (29, 43–46). In central neurons, SK channels contribute to spike frequency adaptation, a process that protects neurons against excessive firing (12, 29, 47–63). Here we demonstrate that SK2 channels are expressed in bladder sensory neurons and that the overexpression of SK2 increases bladder capacity and lowers voiding frequency. In the setting of chemical cystitis, the overexpression of SK2 ameliorates nociceptive behavior. These findings reveal a pivotal role for SK2 channels in normal bladder function and visceral nociceptive signaling and shed further light on their potential as a therapeutic target to treat bladder hyperactivity and visceral pain.

Our studies revealed that the afferents from SK2-KO mice exhibit a steeper response to bladder distension than those of control mice. It has been shown that bladder afferent discharge increases in response to non-voiding transient contractions of the bladder smooth muscle (64), and that apamin increases non-voiding transient bladder contractions and afferent discharge (64). However, deletion of SK2 channels in mice does not result in an increase in bladder basal spontaneous contractility or nerve-mediated contractions (65). Thus, the steeper response of afferents to bladder distention seen in SK2-KO mice cannot be attributed to changes in smooth muscle function. Our FISH studies showed that *Kcnn2* is only expressed in smooth muscle cells in the muscularis externa within the bladder wall; *Kcnn2* expression correlated strongly with *Acta2* expression. Thus, it is unlikely that the effects on afferent firing seen in SK2-KO mice are mediated by other cell types that express SK2 channels. SK channels are known to regulate the firing of neurons by triggering spike frequency adaptation, but they are not considered mechanosensitive per se. Consistent with the latter, the deletion of SK2 does not change the firing threshold of bladder afferents. In summary, our studies suggest that SK2 channels influence the mechanical response of bladder afferents to distension by controlling the rate of firing.

There are conflicting reports regarding the function of SK channels in sensory neurons; some studies suggest that SK channels contribute to the afterhyperpolarization phase (66–68), while others have shown that apamin, a SK inhibitor, has a negligible effect or no effect on this phase of the action potential (12, 69, 70). Our patch-clamp studies showed that apamin increased the firing of a subgroup of bladder-innervating sensory neurons with TTX-R action potentials but, unexpectedly, decreased the firing of those with TTX-S action potentials. Moreover, we did not observe a change in the afterhyperpolarization phase of the action potential of TTX-R or TTX-S bladder sensory neurons with apamin (data not shown). Further studies are needed to understand how SK2 channels regulate bladder afferent discharge.

Voided volumes of SK2^+/T^ mice are nearly 2-fold larger than those of WT littermates. Previous studies have shown that the conditional overexpression of SK3, a subunit expressed in the urothelium and detrusor smooth muscle, promotes structural and functional changes in the bladder (71). Transgenic SK3 mice produce more urine than controls, which is known to cause bladder structural changes in mice and rats with diabetes or receiving chronically diuretics (72, 73). While we cannot rule out the possibility that SK2 overexpression causes structural or molecular changes in the bladder that impact function, urine production (i.e., total voided volume) was similar between SK2^+/T^ and WT littermate mice, and histological sections of SK2^+/T^ mouse bladder showed no obvious structural changes (not shown). Moreover, wall mechanics, elasticity, and apparent bladder capacity of isolated bladder were comparable between SK2^+/T^ and WT littermate mice. Thus, we posit that the increased capacity to store urine seen in SK2^+/T^ mice is a consequence of the overexpression of SK2 in the neuronal circuitry that controls bladder function. Taken together, our studies suggest that SK2 agonists should be considered for the treatment of overactive bladder conditions.

Afferents undergo a wide range of molecular changes in response to tissue injury or inflammation, which alter their threshold for activation and response to physiological stimuli (74). Because of this, there is a surge in afferent drive in pathological states that promotes the sensitization of spinal dorsal horn neurons, a process referred as to “central sensitization,” which is responsible for the hypersensitivity to normally innocuous stimuli and the expanded receptive field seen in acute and chronic visceral pain conditions (4). We found that SK2 overexpression averts the changes in pelvic sensitivity triggered by CYP in WT mice. Bahia and colleagues previously examined the role of SK channels in sensory pathways using pharmacological tools (12). Recordings of lamina V–VI wide-dynamic-range neurons in response to physiological and noxious stimuli provide evidence that SK channels modulate spinal sensory input (12). The exacerbated VMR to bladder distension and pelvic allodynia observed in this study in control mice treated with CYP are consistent with the sensitization of spinal cord circuits receiving nociceptive information from the bladder. While previous reports documented afferent sensitization within 24 hours of the administration of CYP (100 mg/kg) (75, 76), we did not observe significant changes in afferent activity between SK2^+/T^ and WT littermates a week after the initiation of the CYP treatment. We hypothesize that while CYP treatment initially triggers an increase in afferent drive in WT mice, subsequent long-term pain-like behavior is maintained by sensitized central pathways. SK2 is expressed in smooth muscle cells in the bladder wall, in primary afferent neurons, and in neurons of the spinal cord, brainstem, and brain (12, 13). Because in this study we used transgenic mice that overexpressed SK2, we cannot draw conclusions about the site where, or the mechanism by which, SK2 overexpression attenuates pelvic pain. It is unlikely that the difference in visceral pain between SK2^+/T^ and control mice treated with CYP is mediated at the afferent level, because *Kcnn2* expression is comparable in both groups. Thus, future studies are needed to understand the location and actions of SK2 in visceral nociceptive pathways.

Over the past few decades a number of non-opioid agents were developed to treat pain that target voltage-gated Na^+^, K^+^, and Ca^2+^ channels, ligand-gated channels involved in the transduction of sensory information, G protein–coupled receptors, enzymes, and signaling molecules (for details see ref. 10). Yet a relatively low number of candidate drugs developed for pain management ultimately gain FDA approval. Because SK2 channels are closed under basal conditions, pharmacological activators cause a sustained large increase in SK currents. Therefore, we assume that pharmacological activation of SK2 channels will lead to a sustained increase in SK2 activity and blunt pain in cystitis. To date, only a relatively small number of compounds and toxins targeting SK channels, whether activators or blockers, have been identified, and they show limited selectivity among the different isoforms (11). Notably, SK isoforms share a relatively well-conserved transmembrane core, but the N- and C-terminal regions differ markedly (15). These structural differences may be exploited to create more selective agents for SK channel modulation, potentially advancing treatments for overactive bladder and visceral pain.

In summary, the present results demonstrate that a potentiation of SK2 function by means of overexpression ameliorates pain-like behavior in mice subjected to chemical cystitis. SK2^+/T^ mice have no overt physical or behavioral abnormalities compared with WT littermates (31), suggesting a low likelihood of off-target effects in therapeutic applications focusing on the activation of this channel. These results provide important insights into the contribution of SK2 channels to afferent signaling and nociception, highlighting the need for the development of drugs targeting this channel.

## Methods

### Sex as a biological variable.

Our study exclusively examined female mice because the disease modeled is more prevalent in females. The findings of this work might be relevant to both sexes.

### Reagents.

All chemicals were purchased from MilliporeSigma unless otherwise specified.

### Mice.

SK2-KO [B6.129(Cg)-Kcnn2^tm1.1Jpad/J^] (29), SK2^+/T^ [B6.129S4(Cg)-Kcnn2^tm2Jpad^/J] (31), and C57BL/6J mice were obtained from The Jackson Laboratory. Mice were harem-bred and housed in standard cages at the University of Pittsburgh under 12-hour light/12-hour dark cycles with free access to food and water. Experimental mice were 2- to 6-month-old virgin females and were group-housed after weaning up to 5 per cage. The stage of the estrous cycle was not monitored. Mice were randomly assigned to control and treatment groups. Euthanasia was performed using CO_2_ inhalation, followed by thoracotomy as a secondary method.

### Genotyping.

A small piece of tissue was collected from the tail tip and placed in a 1.5 mL tube for storage at –20°C. Genomic DNA was extracted with the hot sodium hydroxide and Tris (HotSHOT) method (77). PCR reactions included 1 μL of sample, 10 μL of 2× GoTaq Green Master Mix (Promega, catalog M7122), 1 μL of DMSO, and primers at a final concentration of 500 nM in a total reaction volume of 20 μL. Cycling conditions were 94°C for 2 minutes, followed by a 10-cycle loop of 94°C for 30 seconds, 65°C (–0.5°C every cycle) for 1 minute, and 72°C for 40 seconds, then a 28-cycle loop of 94°C for 30 seconds, 60°C for 1 minute, and 72°C for 40 seconds, and finally an extension step of 72°C for 5 minutes. The following primers were used for genotyping of SK2-KO mice: common, ATAACCTGTCCCTGCTGCTC; wild-type reverse, GTGGCACTGGTGGTACTGG; mutant reverse, GTAGGTCAGGGTGGTCACGA. The following primers were used for genotyping of SK2^+/T^ mice: common, GCCTCCCAGTACCACCAGT; wild-type reverse, AGAGCGCCAGGTTGTTAGAA; mutant reverse, GGAGTACTCACCCCAACAGC. PCR products were resolved in 2% agarose gels containing ethidium bromide (1 μg/mL) (Bio-Rad, catalog 1610433).

### Retrograde labeling of bladder sensory neurons.

Bladder dorsal root ganglia (DRG) neurons were labeled with the fluorescent retrograde axonal tracer DiI (1,1′-dioctadecyl-3,3,3′,3′-tetramethylindocarbocyanine perchlorate; Thermo Fisher Scientific, catalog D282), cholera toxin β subunit (CTb) conjugated to Alexa Fluor 555 (Thermo Fisher Scientific, catalog C34776), or unconjugated CTb (MilliporeSigma, catalog C9903). Mice were anesthetized with isoflurane, and an abdominal incision was made to expose the bladder. Then, 3–5 μL of DiI (5% wt/vol in DMSO) or CTb (0.1% in sterile saline) was injected at 3–4 locations in the bladder wall with a syringe suited with a 28-gauge needle. After each injection, the needle was kept in place at the site for 20–30 seconds. The muscle layer and skin incisions were closed in layers using a 5.0 PDO absorbable monofilament surgical suture (AD Surgical, catalog S-D518R13). Mice were given ketoprofen (5 mg/kg; Zoetis, Ketofen) subcutaneously for pain relief and ampicillin (100 mg/kg; Eugia US LLC, catalog NDC 55150-113-10) to prevent postoperative infections. Mice were housed under the conditions described above between 7 and 10 days before any further procedure was performed.

### Isolation of bladder sensory neurons.

Lumbosacral (L6–S2) DRG were collected from 2–3 mice and transferred to a cell culture dish containing Neurobasal-A Medium (Thermo Fisher Scientific, catalog 10888022). DRG were cut into 2–3 pieces with a small scissors and then transferred to a cell culture flask containing 5 mL of Neurobasal-A Medium supplemented with 10 mg of collagenase type 4 (Worthington Biochemical, catalog LS004188) and 5 mg of trypsin (Worthington Biochemical, catalog LS003703). The cell culture dish flask containing the tissues was agitated in an incubating rocker (Fisher Scientific, catalog 02-217-761) for 30 minutes at 37°C. Tissue fragments were gently triturated with a fire-polished glass pipette, and the cell suspension was centrifuged at 460*g* for 5 minutes at room temperature in a Sorvall ST8 centrifuge (Thermo Fisher Scientific, catalog 75-200-709). The centrifugation and resuspension steps were repeated 3 times. The pellet from the final centrifugation was resuspended in 1.5 mL of complete Neurobasal-A Medium (Neurobasal-A Medium supplemented with 5% of B27 supplements [Thermo Fisher Scientific, catalog 17504044], 0.5 mM l-glutamine [Thermo Fisher Scientific, catalog A2916801], 100 U/mL of penicillin, 100 μg/mL of streptomycin, and 100 ng/mL nerve growth factor [Thermo Fisher Scientific, catalog 13290-010]). The cell suspension was plated on coverslips (World Precision Instruments, catalog 502041) coated with ornithine (50 mL; Sigma-Aldrich, catalog P4957) and laminin (Thermo Fisher Scientific, catalog 23017-015) inside a 6-well tissue culture plate. After an incubation of 2 hours at 37°C in an atmosphere with 5% CO_2_, 3 mL of warm complete Neurobasal-A Medium was added to each well, and the tissue culture plate was returned to the incubator.

### Analysis of gene expression by RT-PCR.

RNA from lumbosacral (L6–S2) DRG was isolated with the RNAqueous-4PCR kit (Thermo Fisher Scientific, catalog AM1914) according to the manufacturer instructions. Reverse transcription was performed with an AccuScript PfuUltra II RT-PCR kit (Agilent, catalog 600184) using random primers and following the manufacturer instructions. The following primers were used for cDNA amplification by PCR: *Kcnn1* forward, AACTTCCTGGGAGCCATGTG; *Kcnn1* reverse, ACACTTCGGAGCTTCTGAGC; *Kcnn2* forward, TGGATCTGGCAAAGACCCAG; *Kcnn2* reverse, CAGAACCCGGATAACGCTGA; *Kcnn3* forward, GCTATCCACCAACTGCGGG; *Kcnn3* reverse, AGGTGGAGCTGATCCCGATA; *Actb* (β-actin) forward, CCAGCCTTCCTTCTTGGGTAT; and *Actb* reverse, GGGTGTAAAACGCAGCTCAG. PCR reactions included 5 μL of sample, 10 μL of 2× GoTaq Green Master Mix, 1 μL of DMSO, and primers at a final concentration of 500 nM in a total reaction volume of 20 μL. Cycling conditions were 95°C for 1 minute, followed by 45 cycles of 30 seconds at 94°C, 1 minute at 56°C, and 1 minute at 72°C, and a final extension step of 8 minutes at 72°C. Negative controls included GoTaq, primers, and DMSO, but not sample. PCR products were resolved in 2% agarose gels containing ethidium bromide (1 μg/mL).

### FISH and immuno-FISH, including image capture.

Expression of *Kcnn2*, *Asic3*, *Calca*, *Acta2*, and *Kccn1a* was examined with the RNAscope Multiplex Fluorescent v2 kit (Advanced Cell Diagnostics, catalog 323100). The following probes were used: *Kcnn2* (catalog 427971-C1), *Acta2* (catalog 319531-C3), *Calca* (catalog 578771-C3), *Asic3* (catalog 480541-C3), and *Kccn1a* (catalog 476251-C2). RNAscope 3-plex Negative Control Probe (catalog 320871) was used as a negative control to detect nonspecific signal. Mice were euthanized by CO_2_ inhalation, and the urinary bladder and DRG were harvested. Tissues were dipped into a container with optimal cutting temperature embedding medium (OCT; Tissue Plus, Fisher Scientific, catalog 23-730-571) and then transferred to a 10 × 10 × 5 mm Tissue-Tek Cryomold (Sakura Finetek USA, catalog 427971) filled with OCT that was later placed in a –80°C freezer. Sections (8–12 μm) were cut with a Leica CM1950 cryostat (chamber temperature –20°C and knife temperature –18°C) and collected on Superfrost Plus microscope slides (Thermo Fisher Scientific, catalog 12-550-15). Slices containing sectioned tissues were allowed to “dry” in the cryostat chamber for 30 minutes, before long-term storage at –80°C.

FISH and immuno-FISH were conducted according to the Advanced Cell Diagnostics protocol, but with some variations. Slides containing sectioned tissue were removed from the –80°C freezer and immediately fixed for 15 minutes at 4°C with neutral-buffered formalin composed of 29 mM NaH_2_PO_4_·H_2_O, 45.8 mM Na_2_HPO_4_, and 4.0% (vol/vol) paraformaldehyde (neutralized to pH 7.2 while preparing a 40% wt/vol stock). For immuno-FISH, sectioned DRG were incubated overnight with a rabbit antibody against CTb (Novus Biologicals, catalog NB100-63067; dilution 1:100) or a goat anti-CTb antibody (MilliporeSigma, catalog 227040; dilution 1:1,000) at 4°C. After primary antibody incubation, slides were incubated in neutral-buffered formalin for 30 minutes at room temperature. For both FISH and immuno-FISH, tissue sections were then incubated with Protease IV for 5 minutes at room temperature. Probes were developed using TSA Vivid Fluorophore 520 (Tocris Biosciences, catalog 7523) and TSA Vivid Fluorophore 570 (Tocris Biosciences, catalog 7526). For immuno-FISH, after the last treatment with horseradish peroxidase blocker, samples were incubated for 30 minutes at room temperature with a Cy3-conjugated goat anti-rabbit secondary antibody (1:1,000) or a secondary donkey anti-goat antibody conjugated with Alexa Fluor 594 (1:1,000). Nuclei were counterstained with DAPI. Tissues sections were mounted using ProLong Gold antifade mountant (Thermo Fisher Scientific, catalog P36934) and cured overnight at room temperature in the dark. The slides were then stored at 4°C. General image capture was performed using a Leica HCX PL APO ×20, 0.75 numerical aperture dry objective and the appropriate laser lines of a Leica Microsystems SP8 Stellaris confocal microscope outfitted with a 405-laser diode and a white light laser. Eight-bit images were collected at 600 Hz using 2-line averages combined with 2 frame averages. Sequential scanning was used to prevent crosstalk between channels. Stacks of images (1,024 × 1,024, 8-bit) were collected using system-optimized parameters for the *z* axis. Images were processed using the 3D visualization package of Leica LASX software and exported as TIFF files. Final figures were assembled in Adobe Illustrator version 29.6.1.

### Quantification of FISH.

Images were analyzed with CellProfiler (Broad Institute) (78, 79). The area of CTb-labeled neurons, Calca-expressing neurons, and the smooth muscle labeled with Acta2 was defined using the Identify Primary Objects tool (http://cellprofiler.org). Subsequently, cluster number and area within the mask were identified using the Measure Object Size Shape tool (http://cellprofiler.org). The area occupied by the clusters was normalized to the total area of the mask.

### Patch clamp.

Experiments were conducted 2–48 hours after plating of DRG neurons on glass-coated coverslips. Recordings were conducted in an open chamber mounted on the stage of a Nikon Ti inverted microscope equipped with a Sedat Quad set (Chroma Technology), a Lambda XL light source (Sutter Instruments), and an ORCA-Flash 2.8 camera (Hamamatsu). DiI-labeled neurons were identified with an ET555/25x excitation filter (Chroma Technology). Recordings were performed at room temperature with a PC-505B patch-clamp amplifier (Warner Instruments) interfaced to a Digidata 1440A (Molecular Devices) acquisition system. Electrical signals were low-pass-filtered at 1 kHz (4-pole Bessel filter) and digitized at 5 kHz. Data were acquired with a PC running pClamp 10.7 (Molecular Devices). Micropipettes with a fire-polished tip and a resistance of 1.5–3 mΩ were used for whole-cell recordings. The pipette filling solution contained (in mM): 145 KCl, 1 MgCl_2_, 0.1 CaCl_2_, 1 EGTA, and 10 HEPES (pH 7.2). The extracellular bath solution contained (in mM): 135 NaCl, 5 KCl, 1 MgCl_2_, 2.5 CaCl_2_, 10 glucose, and 10 HEPES (pH 7.4). Whole-cell access was obtained with amphotericin B (120 μg/mL). Only DiI-labeled neurons with a resting membrane potential more negative than –40 mV and that generated an action potential with a distinct overshoot exceeding 0 mV in response to a depolarizing current were included in the study. To determine the action potential rheobase, which represents the minimum depolarizing current injection necessary to evoke an action potential, a series of 4 ms rectangular pulses of increasing intensity were applied until an action potential was evoked. Sensory neurons were classified based on the sensitivity of the action potential to 1 μM TTX as TTX-R or TTX-S (22).

### Chemical cystitis.

Cyclophosphamide (CYP; 8 mg/mL; Sigma-Aldrich, catalog C0768) was prepared daily in sterile saline and filtered through a 0.45 μm PVDF filter (Whatman, catalog 9913-2504). Mice received either saline or an intraperitoneal injection of solution containing CYP (80 mg/kg) every 48 hours for a week (4 doses). Experimental procedures were conducted 24–36 hours after the final dose of CYP or saline.

### Assessment of pelvic mechanical sensitivity.

Mice were acclimatized in modular cages (Bioseb, catalog BIO-PVF) mounted on a platform with a wire mesh bottom for at least 1 hour before testing. Withdrawal thresholds to von Frey filaments (Touch Test Sensory Evaluators, North Coast Medical, catalog NC12775-99) were determined with the up-down method described by Chaplan and colleagues (40). Briefly, von Frey filaments were applied to the lower abdominal (pelvic) area for 1–3 seconds, with an interval between stimuli of 15 seconds. Testing was initiated with a filament with a calibrated force of 0.16 grams. Abdominal withdrawal, either contraction of the abdominal musculature or postural retraction of the abdomen, or licking or scratching in the pelvic area in response to the stimulus was considered a positive response. After the response threshold was first crossed, 3 additional filaments were applied that varied sequentially up or down based on the animal response. The resulting pattern of positive and negative responses was tabulated, and the 50% response threshold was calculated using the equation:

50% withdrawal threshold (g) = 10^(Xf^^+^^kδ)^/10,000

where Xf represents the value of the final von Frey hair used, k represents the tabular value for the pattern of positive/negative responses (40), and δ represents the mean difference (in log units) between stimuli.

### Real-time void spot assay.

Voiding was evaluated in freely moving mice with a real-time video monitoring system recently described in detail (41). Briefly, the setup consists of an aluminum stand that houses 2 acrylic cages (37 × 25 × 20 cm), each with a UV-transmitting acrylic bottom. The lower compartment of the stand has reflective mirrored walls and houses 2 UV tube lights (ADJ Products, model T8-F20BLB24 24) that evenly illuminate the bottom of the cages. A filter paper is placed on the bottom interior of the cages to allow visualization of urine spots under UV light. Each mouse is monitored by wide-angle cameras (Logitech, model C930e) that are mounted one above the cage housing the animal and another at the base of the stand lower compartment. The streamed video was captured with an Apple iMac computer running SecuritySpy (Ben Software version 5.5.6) at 1 frame per second with a 1,920 × 1,080 pixel resolution. Cages were suited with the following: a Cosmo blotting paper (Blick Art Materials, catalog 10422-1005) used to visualize urine, an igloo-shaped sleeping chamber, an Eppendorf tube used for enrichment, and a dish with standard mouse chow and water in the form of Hydrogel (ClearH_2_O, catalog 70-01-5022). Mice were routinely housed in a facility with 12-hour light/12-hour dark cycles, with 7:00 am being zeitgeber time (ZT) = 0 (start of light cycle). Mice were introduced and acclimated in the voiding cages for at least 1 hour before data collection. Voiding behavior was evaluated during 12 hours from ZT = 11 to ZT = 23 (dark cycle). Voiding events were identified by visual inspection of the recorded videos, and urine spot area was estimated from video images using ImageJ (Fiji). Void spot volumes were calculated by interpolation of the void spot area in a standard calibration curve generated with known urine volumes.

### Visceromotor response to bladder distension.

Mice were anesthetized with isoflurane followed by urethane (1.5 mg/kg subcutaneously; MilliporeSigma, catalog U2500). Body temperature was maintained with a warming system (Stoelting, catalog 53850) suited with a heating pad (Stoelting, catalog 53812). A lateral skin incision was made to expose the abdominal musculature, and Teflon-coated platinum-iridium wires (A-M Systems, catalog 777000) were implanted in the oblique muscle. The incision was covered with low-toxicity silicone adhesive (World Precision Instruments, catalog KWIK-SIL). A 24-gauge catheter (Smiths Medical ASD, catalog 3063) was implanted in the urethra for saline infusion. The urethral catheter was connected to a 4-way connector: one branch led to a pressure transducer (AD Instruments, catalog MLT844) coupled to a bridge amplifier (AD Instruments, catalog FE224), a second port was connected to a stop valve and reservoir with saline, and the third port was connected to a pressure release valve, which facilitated bladder emptying after filling. Hydrostatic pressure was applied by raising of the reservoir with saline to predefined heights. Electromyography (EMG) signals were amplified (10,000 times) and filtered (0.3–1.0 kHz) with a differential amplifier Model 1700 (A-M Systems, catalog 690000). The amplified signal was passed through a Hum Bug Noise Eliminator (Digitimer, catalog D.HUMBUG) to remove 50/60 Hz noise and harmonics. Pressure and EMG signals were captured and digitized at 5 kHz with a PowerLab 4/35 (AD Instruments) and recorded simultaneously in LabChart software version 8.0 (AD Instruments). EMG responses were recorded at distension pressures of 10, 20, 30, 40, 50, and 60 cmH_2_O for 30 seconds with 3-minute intervals between stimuli. At the end of each stimulus, the bladder was emptied using the release valve. The baseline of the electrical signal was empirically determined. Action potentials that crossed the defined threshold were detected with Spike 2 software version 10.19 (Cambridge Electronic Design). The frequency of discharge at a given pressure was calculated for the initial 25 seconds of the pressure stimulus.

### Ex vivo bladder afferent nerve recordings.

Mice were euthanized by CO_2_ inhalation followed by thoracotomy and exsanguination. The bladder, urethra, and associated L6–S1 spinal nerve roots were dissected in cold Krebs solution consisting of (in mM): NaCl 118.5, KCl 4.7, NaHCO_3_ 25, NaH_2_PO_4_ 1.3, MgSO_4_ 1.2, d-glucose 18, and CaCl_2_ 2.5. A cannula was inserted in the urethra, and the ureters were tied close to the urinary bladder wall. The preparation was carefully transferred to a recording chamber equilibrated with warm Krebs solution bubbled with a mixture of 95% O_2_/5% CO_2_ (vol/vol). The solution in the chamber was continuously recirculated, and the temperature maintained at 35°C with an inline heater (Warner Instruments, model SH-27B) coupled to a dual-channel automatic heater controller (Warner Instruments, model TC-344B). The urethral cannula was coupled to a 4-way connector: one branch led to a pressure transducer (World Precision Instruments, catalog BLPR2), a second port was connected to a syringe pump (World Precision Instruments, model SP100iZ) for continuous infusion with Krebs, and the third port was connected to a pressure release valve, which facilitated bladder emptying after filling. Electrical signals were recorded with a differential amplifier Model 1700 (A-M Systems) and were band-pass-filtered at 100–1,000 Hz. The amplified signal was passed through a Hum Bug Noise Eliminator (Digitimer) to remove 50/60 Hz noise and harmonics. To record afferent nerve activity, a spinal root (L6 or S1) was carefully positioned into a bipolar suction electrode (A-M Systems, catalog 573040) suited with a glass pipette. Pressure and nerve signals were recorded at a rate of 25,000 with a CED 1401 Power 3A data acquisition system (Cambridge Electronic Design) interfaced to a PC computer running Spike 2 software (Cambridge Electronic Design). The preparation was equilibrated in the chamber for at least 30 minutes before filling the urinary bladder. To assess the viability of the preparation and reproducibility of the afferent response to filling, urinary bladders were continuously infused at a rate of 15 μL/min to an intravesical pressure of 40 cmH_2_O. Once this pressure was reached, the bladder was emptied through the urethral cannula by manual opening of the release valve. This protocol was repeated 2 additional times with a resting interval of 10 minutes between fillings. The third filling ramp at 15 μL/min was used for analysis. For the experiments conducted with SK1-KO and WT littermates, the baseline of the electrical signal was empirically determined. For the experiments conducted with SK2^+/T^ and control littermates, TTX at a final concentration of 1 μM was perfused into the chamber at the end of the experiment to block nerve activity and determine the electrical signal baseline. Both methods for baseline determination yielded similar outcomes. Action potentials were detected with Spike 2 software version 10.19 using the baseline empirically determined or estimated with TTX. The frequency of firing at a given pressure was calculated for a 5-second interval. Apparent bladder capacity was calculated by multiplying the time needed to reach a pressure of 40 cmH_2_O by the infusion rate (15 µL/min).

### Statistics.

Data are expressed as mean ± SEM (*n*), where *n* equals the number of independent measurements. Parametric or non-parametric tests were used as appropriate. Mechanical thresholds to von Frey filaments are non-parametrically distributed. Thus, the Kruskal-Wallis test followed by Dunn’s multiple-comparison test was used to analyze von Frey data (40). A *P* value less than 0.05 was considered statistically significant. Statistical comparisons were performed with GraphPad Prism 10 (GraphPad Software).

### Study approval.

All animal studies were performed in accordance with relevant guidelines/regulations of the Public Health Service Policy on Humane Care and Use of Laboratory Animals and the Animal Welfare Act and under the approval of the University of Pittsburgh Institutional Animal Care and Use Committee.

### Data availability.

A Supporting Data Values file is provided as supplemental material. Raw data that support the findings of this study are available on request.

## Author contributions

MDC and GMM conceived the study. NM and MDC performed retrograde tracing and patch-clamp studies. GMM, AM, XS, and MDC performed mechanical allodynia and real-time void spot assays. MDC conducted VMR studies. GMM conducted bladder afferent nerve recordings. XS and MDC performed FISH and immuno-FISH experiments including image capture. XS performed immuno-FISH and FISH image analysis. GMM and MDC analyzed data. AM was responsible for maintaining and breeding of mouse colonies. GMM and MDC wrote the paper with input from all authors. All the authors approved the manuscript.

## Conflict of interest

The authors have declared that no conflict of interest exists.

## Funding support

This work is the result of NIH funding, in whole or in part, and is subject to the NIH Public Access Policy. Through acceptance of this federal funding, the NIH has been given a right to make the work publicly available in PubMed Central.

- NIH grants R01DK134431 (to MDC), DK119183 (to Gerard Apodaca and MDC), and DK138907 (to MDC and Gerard Apodaca).
- Instrumentation Program (1S10OD028596).
- Pittsburgh Center for Kidney Research (U54DK137329).
- Urology Care Foundation Research Scholar Award Program (to NM).

## Supplementary Material

Supplemental data

Supporting data values

## Version 1 05/22/2026

Electronic publication
