# Supplementary material for: Overexpression of small-conductance Ca2+-activated K+ channel 2 attenuates pain-like behavior in female mice with cystitis
Source: JCI Insight. 2026 May 22;11(10):e199567. doi: 10.1172/jci.insight.199567 (PMC13232727; doi:10.1172/jci.insight.199567)
Supplement: Supplemental data [file jciinsight-11-199567-s113.pdf]

Supplemental material

Supplemental Figure 1. Overexpression of *Kcnn2* in transgenic SK2 mice. Fluorescence *in situ* hybridization (FISH) confirms higher *Kccn2* expression in *Calca*$^{+}$ DRG neurons of SK2$^{+/T}$ mice than control littermates (WT). No signal was visible with the negative control probe (not shown). (**A**) Confocal images of DRG are shown. FISH was performed with probes for *Kcnn2* and *Calca*. Insets, 4-fold magnification of *Calca*$^{+}$ neurons. (**B**) Quantification of *Kcnn2* expression in *Calca*$^{+}$ neurons. The fraction area of each *Calca*$^{+}$ neuron occupied by *Kcnn2* clusters is shown. Data are shown as the mean ± SEM (WT, 94 cells from 3 mice; SK2$^{+/T}$, 79 cells from 3 mice; Mann-Whitney test, * $p<0.001$).

Supplemental Figure 2. Cyclophosphamide treatment does not alter *Kcnn2* expression in smooth muscle cells or bladder sensory neurons. Wild type (WT) mice received saline (SAL) or cyclophosphamide (CYP) every other day for a week. (**A**-**D**) Cluster analysis using CellProfiler. (**A**) Confocal images of the DRG are shown. Immuno fluorescence *in situ* hybridization (immuno-FISH) was performed in fresh frozen sections of DRG (L6-S2) harvested from mice injected into the bladder wall with cholera toxin β subunit (CTb). A goat antibody anti-CTb and a secondary donkey anti-goat conjugated with AlexaFluor™ 594 were used to identify bladder sensory neurons (red). (**B**) Identification of CTb-labeled neurons using CellProfiler. (**C**) Detection of *Kcnn2* clusters in sensory neurons. (**D**) 4-fold magnification of the identified neuron in box and detected clusters. (**E**) Quantification of *Kcnn2* expression in CTb-labeled bladder sensory neurons of WT mice treated with SAL or CYP. The fraction area of CTb-labeled neurons occupied by *Kcnn2* clusters is shown. Data are shown as the mean ± SEM (SAL, 73 cells from 3 mice; CYP, 66 cells from 3 mice; not statistically significant) (**F**) Quantification of *Kcnn2* expression in bladder muscularis externa of WT mice treated with SAL or CYP. The fraction area of the muscularis externa occupied

by *Kcnn2* clusters is shown. Data are shown as the mean ± SEM (SAL, n=3; CYP, n=3; not statistically significant).

Supplemental Figure 3. *Kcnn2* is not expressed in skeletal muscle. (**A**) Fluorescence *in situ* hybridization (FISH) was performed on cryosections of abdominal skeletal muscle from $SK2^{+/T}$ and wild type (WT) littermate mice. FISH was performed with probes for *Kcnn2* and *Kccn1a*, which encodes the pore forming subunit of the large conductance calcium-activated K^+ channel. Inset, 4-fold magnification of the skeletal muscle. No signal was visible with the negative control probe (not shown). (**B**) Confocal images of the urinary bladder from $SK2^{+/T}$ and WT littermate mice are shown. FISH was performed with probes for *Kcnn2* and *Acta2*. *Acta2* is highly expressed in smooth muscle cells. Inset, 4-fold magnification of the muscularis externa. Representative of two experiments.

Supplemental Figure 4. Measurement of afferent nerve activity and determination of baseline using tetrodotoxin. Representative recording of intravesical pressure, raw nerve activity and afferent discharge from a bladder of a transgenic SK2 ($SK2^{+/T}$) mouse injected with saline. The urinary bladder was infused at a rate of 15 µl.min-1 until the intravesical pressure reached 40 cmH_2O. Bladder infusion initiation is denoted by a red arrow. At the end of the experiment tetrodotoxin (TTX, 1 μM) was perfused through the chamber and an additional filling cycle at a rate of 130 µl.min-1 was conducted to confirm inhibition of nerve activity.

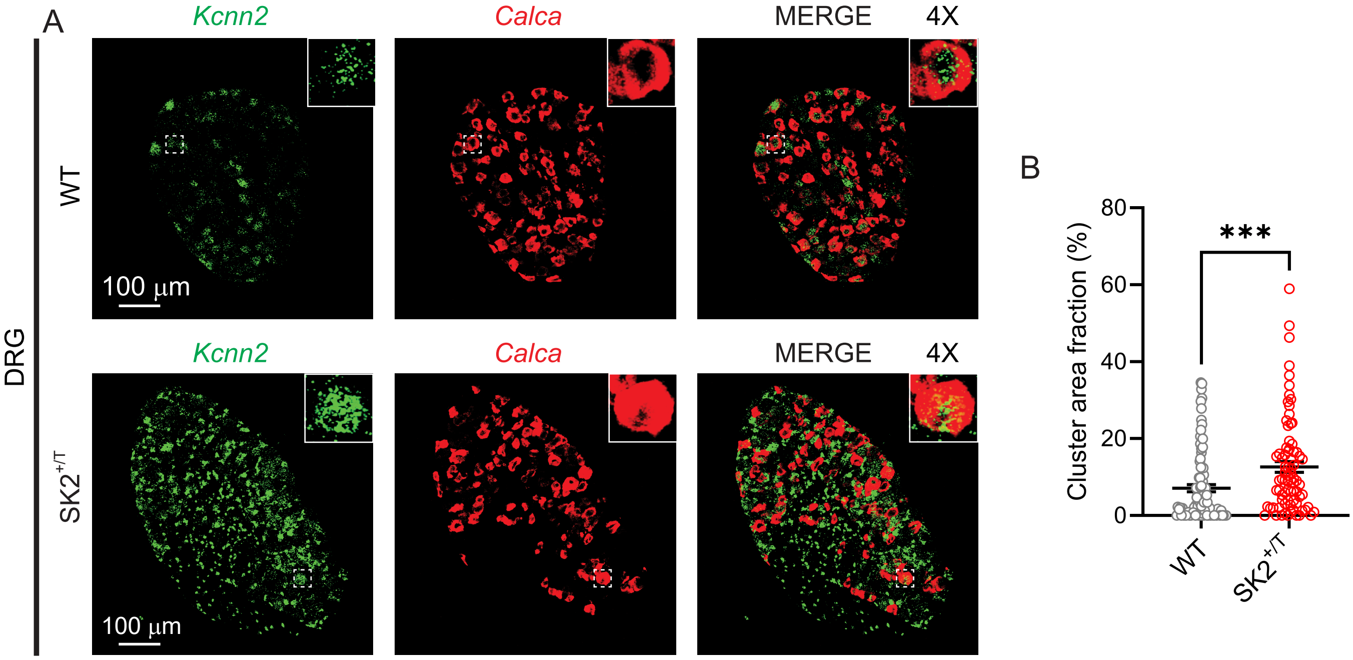

Supplemental Figure 1.

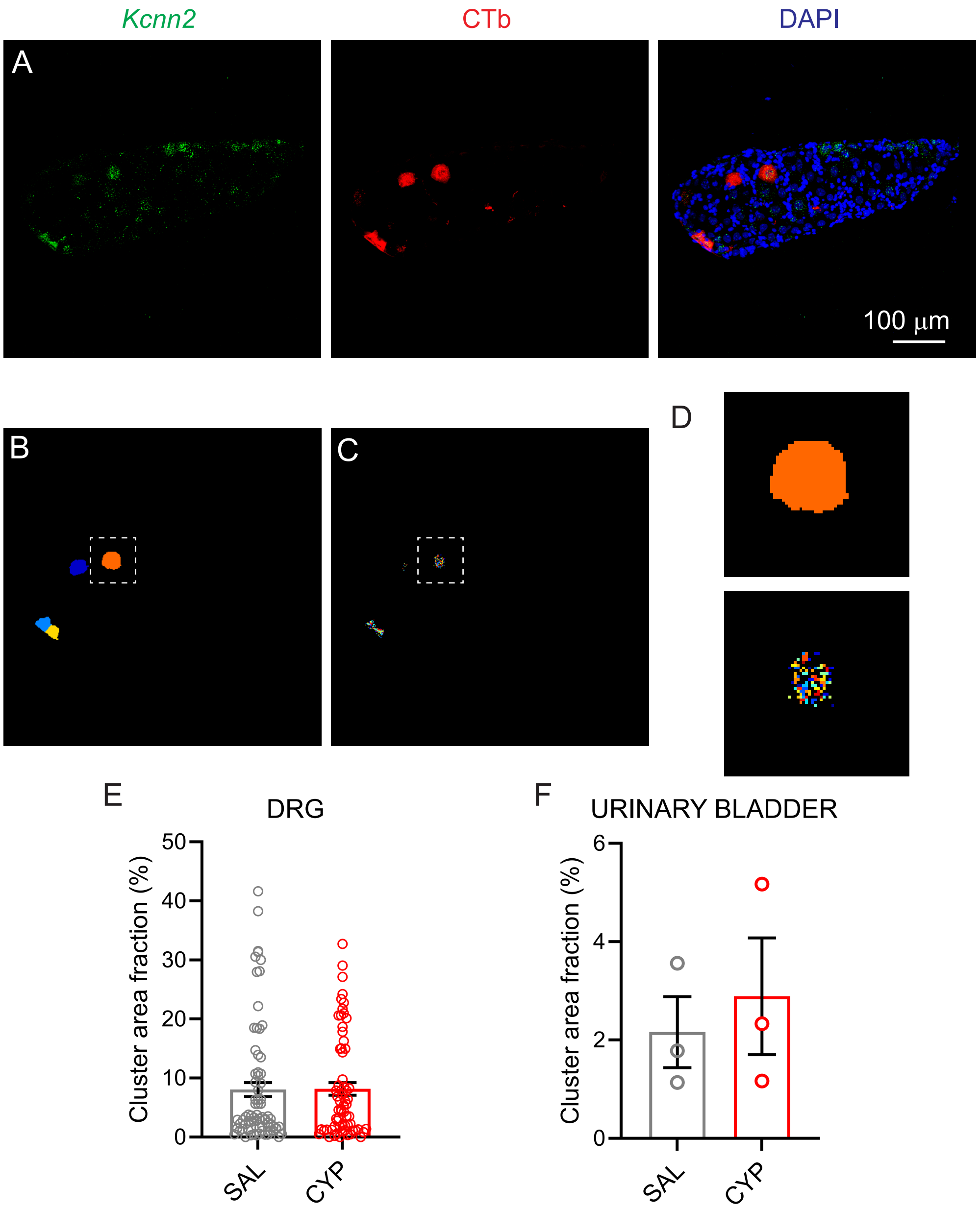

Supplemental Figure 2.

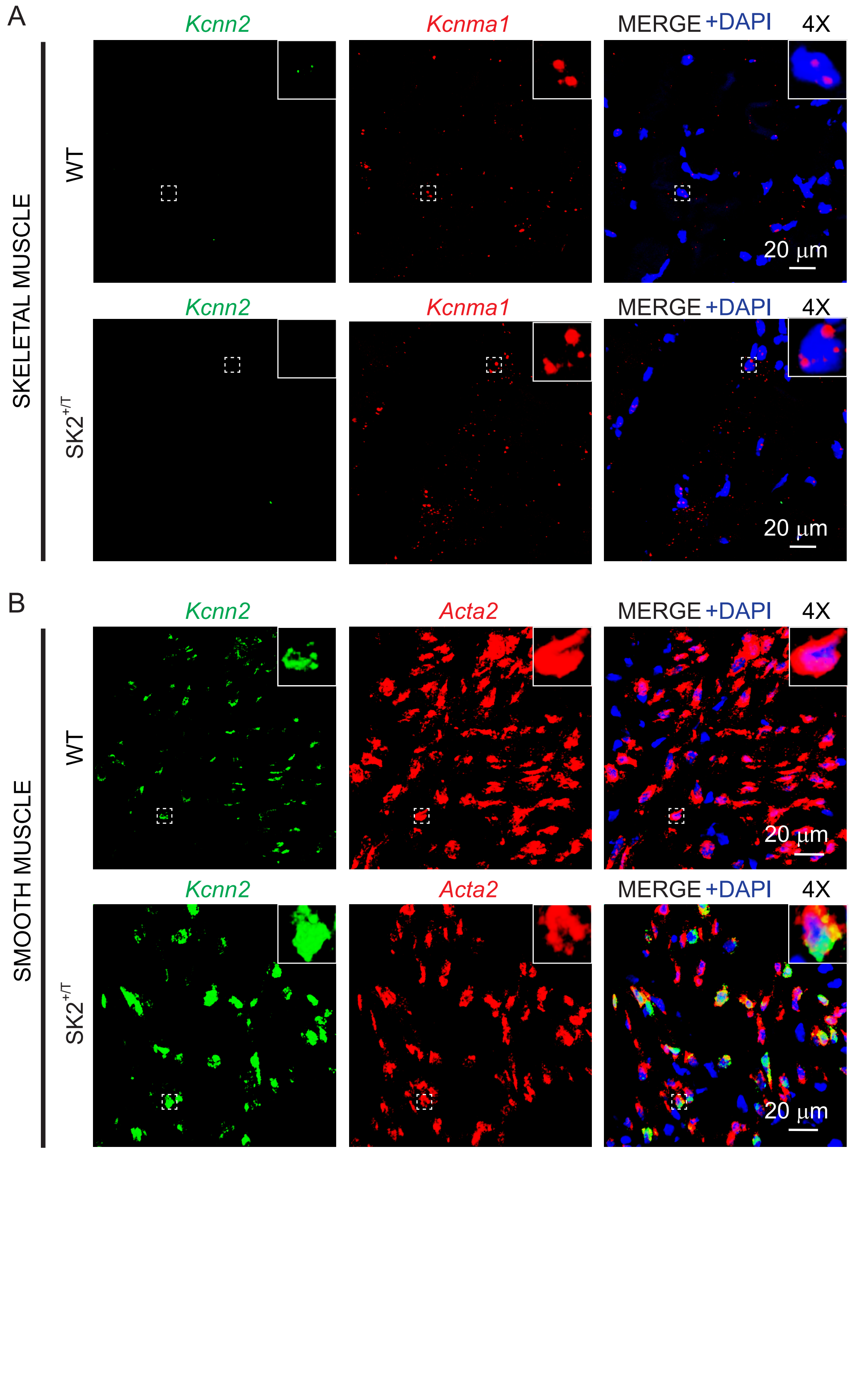

Supplemental Figure 3.

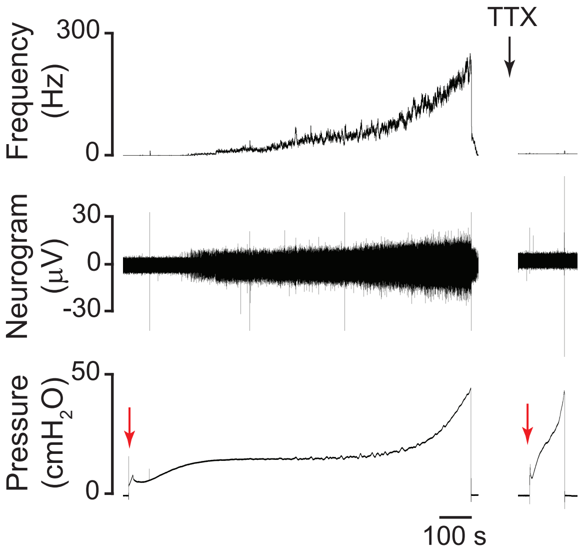

Supplemental Figure 4.
